# Phospholipase C Beta 1: a Candidate Signature Gene for Proneural Subtype High-Grade Glioma

**DOI:** 10.1007/s12035-015-9518-2

**Published:** 2015-11-28

**Authors:** Guangrong Lu, Jeffrey T. Chang, Zheyu Liu, Yong Chen, Min Li, Jay-Jiguang Zhu

**Affiliations:** 1 The Vivian L. Smith Department of Neurosurgery, The University of Texas Health Science Center at Houston (UTHealth) Medical School, 6400 Fannin Street, Suite 2800, Houston, TX 77030 USA; 2 The Department of Integrative Biology and Pharmacology, The University of Texas Health Science Center at Houston (UTHealth) Medical School, Houston, TX 77030 USA; 3 Division of Biostatistics, UTHealth School of Public Health, Houston, TX 77030 USA; 4 Department of Biostatistics and Epidemiology, Center for Clinical Epidemiology and Biostatistics, Perelman School of Medicine, University of Pennsylvania, Philadelphia, PA 19104 USA; 5 Department of Medicine and Department of Surgery, The University of Oklahoma Health Sciences Center, Oklahoma City, OK 73104 USA

**Keywords:** PLCβ1, Glioma, Glioblastoma, Proneural, Signature gene, Biomarker, REMBRANDT, TCGA

## Abstract

Phospholipase C beta 1 (PLCβ1) expresses in gliomas and cultured glial cells, but its expression is barely detectable in normal glial cells. We analyzed data from Gene Expression Omnibus (GEO-GDSxxx), The Cancer Genome Atlas (TCGA), and the Repository for Molecular Brain Neoplasia Data (REMBRANDT) to explore the potential role of PLCβ1 as a biomarker in high-grade glioma (HGG). PLCβ1 expression is significantly higher in grade III gliomas than that in grade IV gliomas from GDS1815 (*n* = 24 vs. 76), GDS1962 (*n* = 19 vs. 81), and GDS1975 (*n* = 26 vs. 59). In GDS1815, PLCβ1 expression correlates with several known proneural (PN) signature genes; its expression from PN subtype (*n* = 15) is significantly higher than that from mesenchymal (Mes) subtype (*n* = 33) HGG. In GDS1962, PLCβ1 expression is the highest in nontumor brain tissue (*n* = 23) and is significantly higher than its expression in grade II gliomas [astrocytomas (*n* = 7) and oligodendrogliomas (*n* = 37)]. A Kaplan-Meier survival curve from a REMBRANDT cohort demonstrates that glioma patients with intermediate PLCβ1 expression (*n* = 103) survived significantly longer than PLCβ1 downregulated (2X) groups (*n* = 226). From TCGA data, PLCβ1 RNA-Seq signal inversely correlates with the pathological grades, and PLCβ1 expression in PN (*n* = 8) is of significantly higher levels than that in Mes (*n* = 8) subtypes of glioblastoma. The top 50 % of PLCβ1 expression subgroup (*n* = 294) of gliomas (grades II to IV merged) survived significantly longer than the low 50 percentile of the PLCβ1 expression subgroup (*n* = 293). *p* values are less than 0.05 for all these analyses. We conclude that PLCβ1 is a candidate signature gene for PN subtype HGG, and its expression inversely correlates with glioma pathological grade and is a potential prognostic factor.

**Electronic supplementary material:**

The online version of this article (doi:10.1007/s12035-015-9518-2) contains supplementary material, which is available to authorized users.

## Introduction

Gliomas are the most common primary brain tumors, and the pathologic grade is the most important factor determining patients’ prognosis [1, 2]. Originally established by the World Health Organization (WHO) in 1993 and then updated in 2007, the four-tiered grading system for glioma outlines grade I as the least aggressive and grade IV as the most aggressive type of gliomas [3]. Grades I/II and III/IV are also collectively referred to as *low-* (LGG) and *high-*grade gliomas (HGG), respectively. Grade IV gliomas, also known as glioblastoma multiforme (GBM), are the most common and the most malignant type of primary brain tumor and account for 50–60 % of all gliomas [4]. Patients can develop gliomas at any age; however, LGGs are more often found in children and young adults and HGGs are more prevalent in the elderly. Other known prognostic factors include patient’s age at initial diagnosis, extent of tumor resection, and Karnofsky performance score [1, 5].

With advancement of molecular biology technology, new methods for classifying gliomas have emerged in recent years [6]. The early established methods include gene mutation analysis and genome-wide screening, which permit identification of single or multiple gene abnormalities, epigenetic changes, and/or chromosomal anomalies [7–9]. The latest developments, including gene expression profiling, microarray technique, and whole genome sequencing, are capable of studying tens of thousands of genes simultaneously. Previous studies have identified groups of genes being up- or downregulated within different subtypes of gliomas. Some gene expression patterns are associated with patient survival rates [6]. As a result, a new concept of “signature” genes has been introduced to classify HGG based on global gene transcripts differences [10–12]. For example, two studies subclassified HGG into three (Mes, PN, and proliferative) and four (Mes, PN, neuronal, and classical) subtypes, respectively [10, 11]. Two different sets of signature genes for PN subtypes were used to separate the PN subtype from the other subtypes: set 1 of the signature genes for PN subtypes comprised OLIG2, MAP2, DCX, NeuN, ERBB4, GAD2, etc. and set 2 consisted of OLIG2, ERBB3, SOX2, NKX2-2, DLL3, etc. [10, 11]. CHI3L1/YKL-40 is used as Mes subtype signature gene by both studies [10, 11]. Other Mes signature genes include HLA-G, SERPINE1, CA12, etc. [11]. Strikingly, the expression of signature genes for PN and Mes subtypes appears to be mutually exclusive. It is observed that during GBM progression or recurrence, a unidirectional subclass shift occurs from PN toward Mes subtype, which represents a possible common pattern of disease progression [11]. Patients with PN subtype GBM survive longer on average than those afflicted with other subtypes despite the fact that PN subtype glioblastoma patients do not benefit significantly from temozolomide and radiotherapy [10, 11]. Although studies attempting to classify GBM subtypes do not always use the same set of signature genes, classification of PN and Mes subtypes is concordant in almost all related studies [10, 11, 13]. Therefore, it is desirable to identify biomarkers that allow quick and accurate identification of PN from other subtypes.

The phospholipase C beta 1 (PLCβ1) gene is mapped to human chromosome 20p12. It is expressed predominantly in neurons of the central nervous system and is barely detectable in other tissues or cell types [14–17]. The PLCβ1 protein catalyzes the formation of inositol 1,4,5-trisphosphate and diacylglycerol from phosphatidylinositol 4,5-bisphosphate (PIP2), which plays an important role in the intracellular signal transduction of extracellular signals, such as metabotropic glutamate. In rodent brain tissue, PLCβ1 is present in pyramidal neurons and interneurons, but is absent in astrocytes by immunohistochemistry [15, 18, 19]. However, PLCβ1 expression is detectable in cultured oligodendrocytes and astrocytes [20, 21]. In addition, PLCβ1, while absent in freshly isolated normal glial cells, has been detected in C6 rat glioma cell lines [21–23].

We hypothesize that PLCβ1 could be a signature gene in glioma subclassification given its absence in normal glial cells, yet presence in glioma specimens. In addition, PLCβ1 expression is inducible in primary cultured glial cells [21, 22].

Microarray and RNA Sequencing (RNA-Seq) data, including that measuring PLCβ1 expression, was submitted to the NIH-maintained Gene Expression Omnibus (GEO) and The Cancer Genome Atlas (TCGA) database [11, 12, 24]. In addition, GEO dataset-GDS1815, the Repository for Molecular Brain Neoplasia Data (REMBRANDT, https://caintegrator.nci.nih.gov/rembrandt), and TCGA databases provide patient survival information along with PLCβ1 microarray/RNA-Seq data, both of which allow investigators to perform survival analysis based on different PLCβ1 expression levels. In this study, we retrieved data and performed analyses to determine (1) if PLCβ1 is a candidate signature gene for the PN subtype glioma, (2) the potential correlation between its expression and glioma’s pathological grades, and (3) its prognostic value in glioma patients.

## Materials and Methods

### Ethics Statement

The usage of data and images from the NIH- and TCGA-maintained databases and from the Human Protein Atlas (HPA, http://www.proteinatlas.org) in this study meets the data use policies set by NIH, TCGA, and HPA, respectively.

### Access to Online Public Databases

For this study, we used online public databases containing PLCβ1 microarray and RNA-Seq data, survival data, and immunohistochemistry (IHC) results. Microarray data is accessible from NIH-maintained website GEO, which serves as a public repository for a wide range of high-throughput experimental data. These datasets include single and dual channel microarray-based experiments measuring messenger RNA (mRNA) data. GEO staffs have selectively curated GEO series into a more compact format, GEO dataset (GDSxxxx), which includes a single spreadsheet of “final” values and accompanying rich sample annotation. Original microarray data can be accessed through links provided in Table 1. Three PLCβ1 probes (213222_at; 215687_x_at; 211925_s_at) are used in datasets of GDS1815, GDS1962, and GDS1975, which studied 100, 180, and 85 cases, respectively [11, 12, 25, 26]. GDS2853 dataset used a different probe, 35980_at PLCβ1 [27].

**Table 1:** Microarray data retrieved from NIH-maintained GEO databases in this study

Database	Contributor	Samples	Probes	Link
GDS1815 [11, 25]	Genentech, Inc., South San Francisco, CA, USA	100 cases Grade III and IV high-grade gliomas; primary and recurrent tumors	PLCβ1-1	http://www.ncbi.nlm.nih.gov/geo/tools/profileGraph.cgi?ID=GDS1815:213222_at
			PLCβ1-2	http://www.ncbi.nlm.nih.gov/geo/tools/profileGraph.cgi?ID=GDS1815:215687_x_at
			PLCβ1-3	http://www.ncbi.nlm.nih.gov/geo/tools/profileGraph.cgi?ID=GDS1815:211925_s_at
			OLIG2	http://www.ncbi.nlm.nih.gov/geo/tools/profileGraph.cgi?ID=GDS1815:213825_at
			ERBB4	http://www.ncbi.nlm.nih.gov/geo/tools/profileGraph.cgi?ID=GDS1815:214053_at
GDS2853 [27]	Children’s National Medical Center, Washington, DC, USA	14 cases Low- vs. high-grade astrocytomas	PLCβ1	http://www.ncbi.nlm.nih.gov/geo/tools/profileGraph.cgi?ID=GDS2853:35980_at
			OLIG2	http://www.ncbi.nlm.nih.gov/geo/tools/profileGraph.cgi?ID=GDS2853:40624_at
			ERBB4	http://www.ncbi.nlm.nih.gov/geo/tools/profileGraph.cgi?ID=GDS2853:37273_at
GDS1962 [26]	National Cancer Institute, Bethesda, MD, USA	180 cases Nontumors, astrocytoma (grades II, III, IV), oligodendroglioma (grades II and III)	PLCβ1	http://www.ncbi.nlm.nih.gov/geo/tools/profileGraph.cgi?ID=GDS1962:213222_at
GDS1975 [12]	Translational Genomics, Phoenix, AZ, USA	85 cases Astrocytomas, mixed oligoastrocytoma, oligodendroglioma, and GBM	PLCβ1	http://www.ncbi.nlm.nih.gov/geo/tools/profileGraph.cgi?ID=GDS1975:213222_at

We also acquired LGG and HGG data (RSEM-normalized RNA-Seq data) from the Broad Firehose database released on December 6, 2014 by TCGA. Data from glioma grades II to IV was merged for analyzing PLCβ1 gene expression; batch effects were removed by using the distance-weighted discrimination (DWD) method [28]. From the clinical annotations, we extracted the pathological grades, patient survival status, and censored data. Glioblastoma subtypes (PN, Mes, classic, and neuronal or proliferative) were previously determined in other studies [10].

The REMBRANDT database is in the public domain and open for researchers. The REMBRANDT website provides different modes to extract raw data for analysis. These selections are glioma and its subcategories consisting of astrocytoma, oligodendrogliomas, and GBM. This study focuses on glioma and its subclass astrocytoma since glioma and astrocytoma data contain samples from three (II, III, and IV) pathological grades, and they are large sample sizes for analysis. Oligodendrogliomas and GBM only have two (II and III) and one (IV) grades, respectively, and their sample sizes are relatively small for analysis. The default setting for Kaplan-Meier survival plot is based on stratified groups of genes upregulated ≥2.0-folds, downregulated ≥2.0-folds, and at the intermediate level; reporter type is Affymerix. For a survival plot based on PLCβ1 gene expression, the default setting selects data generated from the 213222_at probe because its data has the highest geometric mean intensity. (*Note:* The REMBRANDT data portal has been migrated to the Georgetown Database of Cancer, a knowledge discovery platform which supports access of the data by the scientific community.)

Human Protein Atlas is a website that enables systematic analysis of the human proteome using antibody-based proteomics and provides images for IHC results in human tissues. This website permits the utilization and publication of its data as outlined in its *Data Usage Policy* (“the publication and/or presentation are solely for informational and non-commercial purposes” and “the source of the data and/or image is referred to this site and/or one or more of our publications are cited.”)

### Antibody Used in IHC

Based on the information on the HPA website, glioma tissue was stained with four different PLCβ1 antibodies [HPA034743 and HPA057910 (Sigma-Aldrich, St. Louis, MO); CAB004275 and CAB005334 (Santa Cruz Biotechnology, Dallas, TX)]. Patient samples were all deidentified and subjects are listed by ID, gender, age, and pathological grade.

### Statistical Analysis

All data is presented as mean values ± standard error of the mean (SEM) in the histograms. Distribution of expression level was examined by quantile-quantile plot (Q-Q plot) to compare with normal distribution. Statistical association was measured by Pearson correlation embedded in Microsoft Excel. GraphPad Prism (version 6.0; GraphPad Software, San Diego, CA, USA) was used to generate both a bar graph histogram and a Kaplan-Meier survival plot. We compared the expression level of PLCβ1 across grades or subtypes with a two-tailed *t* test; the log-rank test was used for the Kaplan-Meier survival plot. A difference was considered to be statistically significant if the *p* value was less than 0.05 (i.e., *p* < 0.05) [29].

## Results

### PLCβ1 Expression Correlates with Other Known PN Subtype Signature Genes in GDS1815

The GDS1815 dataset contains microarray data generated from 22,283 gene probes in 100 high-grade glioma samples. Three probes (213222_at; 215687_x_at; 211925_s_at) were used to detect PLCβ1 transcripts. Microarray data, presenting as signal strengths generated by probe 213222_at, is highly correlated with other two groups of PLCβ1 microarray data generated by probes 215687_x_at and 211925_s_at. Correlation scores between microarray data of 213222_at and 215687_x_at and microarray data of 213222_at and 211925_s_at are 0.73099 and 0.79047, respectively (Table 2 and Supplement Excel file). Data obtained by the 213222_at probe showed the highest geometric intensity and was chosen for our data analysis; the REMBRANDT database also uses this data as default for the Kaplan-Meier survival analysis. PLCβ1 microarray signal strength also yields a good correlation coefficient with other PN signature genes including DLL3 (*r* = 0.5), HEY2 (*r* = 0.5), Olig2 (*r* = 0.48), BCAN (*r* = 0.62), and ERBB4 (*r* = 0.62) [11]. PLCβ1 microarray signal strength, however, inversely correlates with YKL-40 (*r* = −0.59), one commonly used mesenchymal cell marker in dataset GDS1815.

**Table 2:** Correlation coefficient between PLCβ1 expression and other signature genes [11] in GDS1815

Genes	Probes	Coefficient with PLCβ1 probe 213222_at (*r* =)
PLCβ1	213222_at	1
	215687_x_at	0.73
	211925_s_at	0.79
DLL3	219537_x_at	0.50
HEY2	219743_at	0.50
Olig2	213825_at	0.48
	213824_at	0.37
BCAN	219107_at	0.54
	221623_at	0.64
	91920_at	0.53
ERBB4	214053_at	0.62
	206794_at	0.53
YKL-40	209395_at	−0.47
	209396_s_at	−0.59
	216546_s_at	−0.30

### PLCβ1 Microarray Data Differentiates PN Subtype from Other HGG Subtypes in GDS1815

**Fig. 1:**
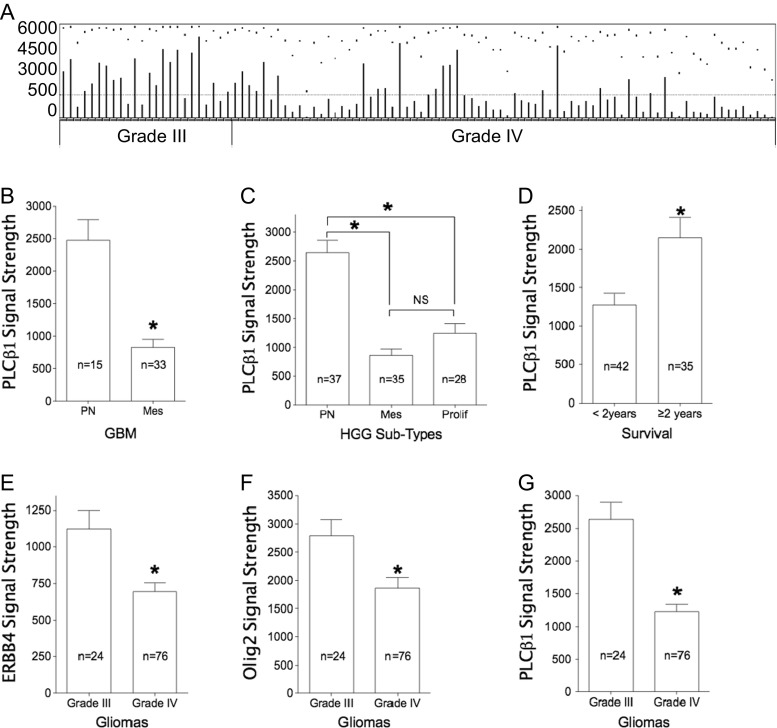
**a** Original microarray data showing PLCβ1 signal strength in GDS1815 dataset from the NIH website with minor modification. **b** Analysis of data from grade IV samples, primary and recurrent tumors combined. Data shows a significant lower level of PLCβ1 in Mes (*n* = 33) subtypes than in PN (*n* = 15; *p = 1.4E-4). **c** Analysis of combined samples of grades III and IV shows that the average PLCβ1 signal level in PN subtypes (2642 ± 207, *n* = 37) is significantly higher than its level in Mes (852 ± 120, *n* = 35; *p* = 2.78E-10) and proliferative (Prolif) subtypes (1242 ± 170, *n* = 28; *p* = 5.14E-6, *p < 0.05). The difference between the PLCβ1 signal level in the Mes and proliferative subtype HGG is not statistically significant (*p* = 0.126). *NS* nonsignificant. **d** In primary tumor cases, pooled data from grade III and grade IV gliomas shows that PLCβ1 microarray signal strengths are significantly higher in patients who survived over 2 years (mean = 240.3 weeks, range 106–477 weeks, *n* = 35) than those survived less than 2 years (mean = 55.4 weeks, range 3–102 weeks, *n* = 42; *p = 0.0035; 23 cases do not have survival data). **e**–**g** ERBB4, Olig2, and PLCβ1 expression, respectively. All of these signal strengths are significantly lower in grade IV glioma samples (*n* = 76) than those in grade III gliomas (*n* = 24; *p < 0.05)

Raw data histogram of one PLCβ1 probe (213222_at) is reproduced from the NIH website (Fig. 1a). The average PLCβ1 signal strength is significantly lower in Mes (*n* = 33) than in PN subtypes (*n* = 15) among GBM (*p* < 0.001; Fig. 1b). Furthermore, PLCβ1 expression results can potentially identify PN and Mes subtypes, regardless of whether samples are from primary or recurrent GBMs (Supplement data Figs. S1A and S1B). In combined samples of grade III and IV gliomas, the average PLCβ1 signal level in PN subtypes (2642 ± 207, *n* = 37) is still significantly higher than its level in Mes (852 ± 120, *n* = 35) and in proliferative subtypes (1242 ± 170, *n* = 28; both *p* < 0.05). However, PLCβ1 content shows no statistical difference between Mes and proliferative subtypes (*p* > 0.05; Fig. 1c).

### Validation of PLCβ1 as a PN Subtype Signature Gene in the TCGA Cohort

**Fig. 2:**
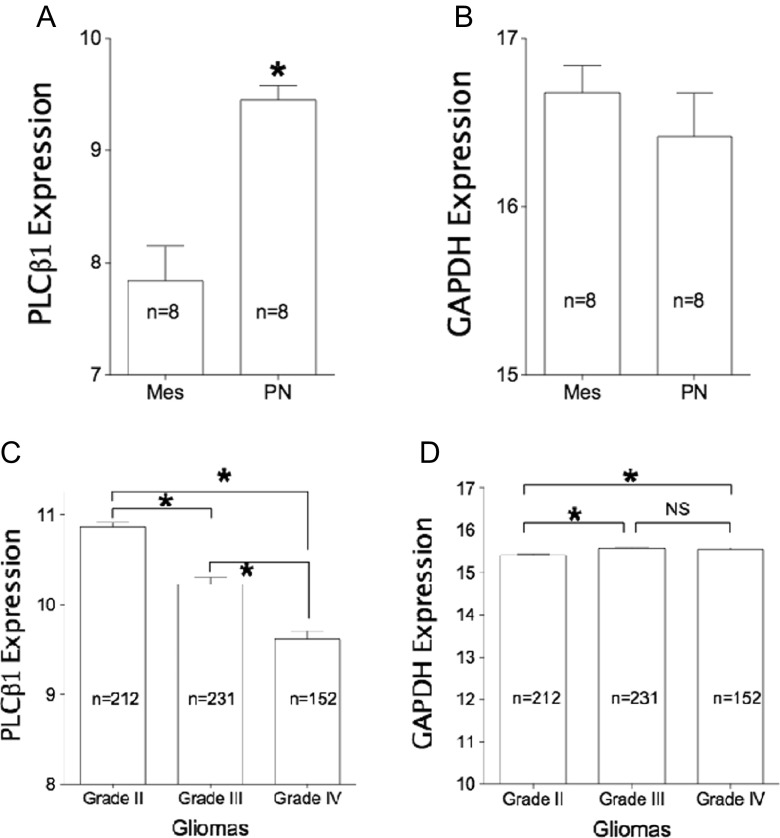
HGG in TCGA only has a few cases that are labeled as Mes and PN subtypes. **a** Normalized PLCβ1 expression (RNA-Seq data) is significantly higher in PN (*n* = 8) than in Mes subtypes (*n* = 8; *p = 4E-4). **b** In contrast, there is no statistical difference in GAPDH expression between PN and Mes subtypes (*p* = 0.41). **c** In the TCGA database, normalized PLCβ1 expression (RNA-Seq data) shows significant differences between grades II (10.87 ± 0.05; *n* = 212), III (10.23 ± 0.08; *n* = 231), and IV (9.61 ± 0.10; *n* = 152; *p < 0.0001). **d** GAPDH expression shows no significant difference between grades III (15.56 ± 0.03) and IV (15.54 ± 0.05; *p* = 0.68). *NS* nonsignificant. However, grade II gliomas (15.41 ± 0.03) contain less GAPDH than grade III (*p* = 0.017) and grade IV (*p* = 0.0018; *p < 0.05)

In GBM cases from the TCGA cohort, PLCβ1 expression (RNA-Seq data) is significantly higher in the PN (*n* = 8) than that in the Mes subtype (*n* = 8; *p* < 0.05; Fig. 2a). While the difference between glyceraldehyde 3-phosphate dehydrogenase (GAPDH) expression levels in PN and Mes subtypes (Fig. 2b) is not statistically significant, GAPDH gene expression is commonly used as an endogenous control.

### PLCβ1 Expression Inversely Correlates with Glioma Pathological Grades

Known PN subtype signature genes are not only useful in separating PN from Mes subtypes, but their expression levels also correlate with different grades of tumors. Both microarray data of ERBB4 (Fig. 1e) and Olig2 (Fig. 1f), two known PN subtype signature genes, show significantly different expression levels between grade III and IV gliomas in GDS1815 dataset (*p* = 0.01 and 0.005, respectively). In agreement with these two signature genes, PLCβ1 (Fig. 1g) also shows a significantly different expression between grade III (*n* = 24) and grade IV (*n* = 76) gliomas (*p* < 0.001).

We further validated the relationship between PLCβ1 expression and gliomas’ pathological grades using other three independent GEO datasets (GDS2853, GDS1962, GDS1975). Specifically, GDS2853 studied low- (*n* = 8) and high-grade (*n* = 6) astrocytomas with 12,625 probes; GDS1962 studied nontumor controls (*n* = 23), grade II (*n* = 7) and III (*n* = 19) astrocytomas, GBM (*n* = 81), grade II (*n* = 37) and III (*n* = 13) oligodendrogliomas with 54,675 probes; and GDS1975 studied grade III [astrocytes (*n* = 8), mixed oligoastrocytoma (*n* = 7), oligodendroglioma (*n* = 11)] and GBM (*n* = 59) with 22,283 probes. DGS1815, GDS1962, and GDS1975 used the same aforementioned three PLCβ1 probes, and we only did analysis of the data generated using probe 213222_at.

**Fig. 3:**
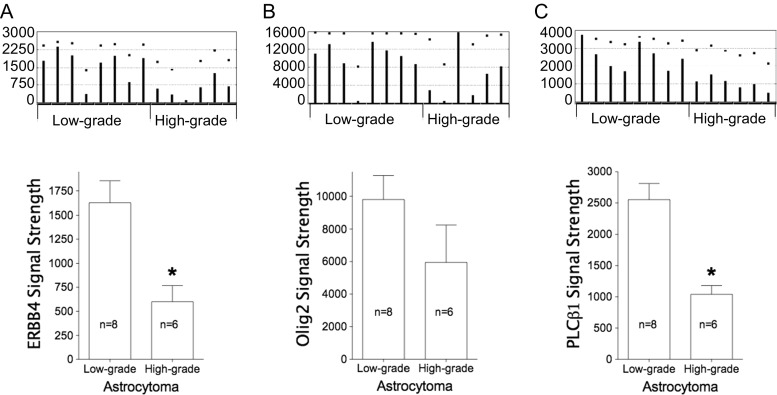
ERBB4, Olig2, and PLCβ1 expressions presented as microarray data in low- and high-grade astrocytomas (*n* = 14). Original GDS2853 data from the NIH-maintained database are on top of each histogram. **a**, **b** ERBB4 and Olig2 microarray data used to do the same analysis; however, the ERBB4 signal was significantly reduced in high-grade samples (*p = 0.0037), but the Olig2 signal (*p* = 0.19) was not. **c** PLCβ1 signal strength among high-grade astrocytoma samples (*n* = 6) significantly decreased in comparison to its signal from low-grade astrocytoma (*n* = 8; *p = 0.0004)

The GDS2853 gene expression profile revealed that the signal strength of the signature gene ERBB4 (Fig. 3a), but not Olig2 (Fig. 3b), was significantly different between low-grade and high-grade astrocytomas; PLCβ1 data analysis agrees with ERBB4 in separating low-grade (*n* = 8) and high-grade (*n* = 6) tumors (Fig. 3c; *p* = 4.0E-4). In comparison, PLCβ1 raw data shows less variation than that seen in ERBB4 data.

**Fig. 4:**
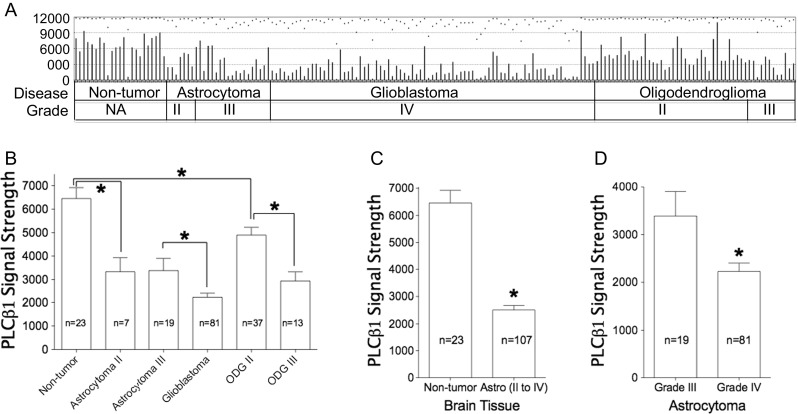
Analysis of PLCβ1 expression profile from GDS1962. **a** Original microarray data copied from the NIH website with minor modification. *NA* nonapplicable. **b** Collective PLCβ1 data analysis. Average PLCβ1 signal from nontumor controls (*n* = 23) is the highest, significantly higher than low-grade tumors including grade II astrocytomas (*n* = 7, *p* = 0.00097) and oligodendrogliomas (*n* = 37, *p* = 0.0075). The average PLCβ1 signal from grade II (*n* = 37) is significantly higher than that from grade III (*n* = 13) oligodendroglioma (*p* = 7.42E-05). **c** PLCβ1 signal levels from nontumor controls (*n* = 23) are significantly higher than those from astrocytomas cases (Astro, *n* = 107, p=9.63E-09), grades II to IV combined. **d** PLCβ1 signal levels from grade III (*n* = 19) astrocytomas are significantly higher than from grade IV astrocytomas (*n* = 81; *p = 0.044)

Among all four GDS datasets, only GDS1962 (Fig. 4a) contained nontumor samples as controls. Figure 4b shows that among all groups, the average PLCβ1 signal strengths were the highest in the nontumor controls (*n* = 23), followed by the grade II (*n* = 7), grade III (*n* = 19), and grade IV (*n* = 81) astrocytoma groups (Fig. 4b). The results that average PLCβ1 expression in grade III (*n* = 19) is significantly higher than in grade IV (*n* = 81) astrocytoma (Fig. 4d; *p* = 0.044) is consistent with previous analysis. The average PLCβ1 expression in nontumor controls (*n* = 23) is also significantly higher than in pooled data of all astrocytoma cases (*n* = 107, *p* = 9.6E-9; Fig. 4c). We also analyzed oligodendroglioma cases separately and found that PLCβ1 expression is inversely related to pathological grades. Grade III (*n* = 13) oligodendroglioma has a significantly lower level than that of grade II (*n* = 37, *p* = 7.0E-5; Fig. S2A). Furthermore, overall oligodendroglioma samples (*n* = 50) showed significantly higher levels of PLCβ1 than astrocytomas (*n* = 107, *p* = 1.4E-6; Fig. S2B). In GDS1975 [12], PLCβ1 expression was also significantly lower in grade IV (*n* = 59) than in grade III gliomas (*n* = 26, *p* = 0.3.0E-6; Fig. S3).

Consistent with the outcomes of data analysis using GEO datasets, PLCβ1 expression level (RNA-Seq data) inversely correlates with pathological grades (II, III, and IV) in the TCGA (Fig. 2c), while GAPDH data shows less significant difference among subtype groups and no significant difference between grades III and IV (Fig. 2d). GAPDH is commonly used as the internal control. Thus, we summarize this inverse relationship between PLCβ1 expression and glioma pathological grades in diagram (Fig. 8).

### PLCβ1 Expression Is Closely Associated with Patient Survival in the REMBRANDT Cohort

**Fig. 5:**
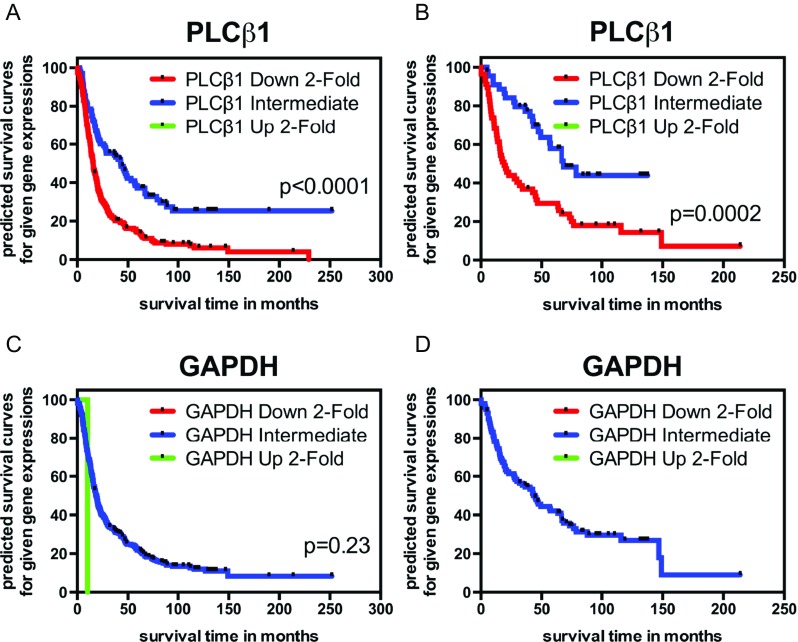
Kaplan-Meier survival curve for samples with differential PLCβ1 and GAPDH gene expression, respectively; raw data extracted from the REMBRANDT cohort. **a** PLCβ1 downregulated glioma patients (*n* = 226) had significantly shorter survival times than intermediate level PLCβ1 glioma patients (*n* = 103) per the log-rank test (*p* = 3.0E-09). **b** PLCβ1 downregulated astrocytoma patients (*n* = 57) survived significantly shorter than the intermediate level PLCβ1 astrocytoma patients (*n* = 45) per the log-rank test (*p* = 2.0E-04). Both databases contain no cases stratified as upregulated PLCβ1 expression (≥2X). **c** Based on GAPDH expression level, only 1 glioma case is classified as upregulated (≥2X), not statistically significant in survival from the group of intermediate level GAPDH (*n* = 328; *p* = 0.23). There are no GAPDH downregulated glioma patients (*n* = 0). **d** Based on the GAPDH expression level, there are no astrocytoma cases being classified as up- (*n* = 0) or downregulated (*n* = 0) exceeding 2-fold. Intermediate level of GAPDH is the only group (*n* = 102). Note: Among the raw data we downloaded for analysis, 14 glioma cases (include 3 astrocytoma) lacked censored information. Thus, our analysis has 14 fewer glioma cases (include 3 astrocytoma) than the total cases reported in the REMBRANDT website (see Table 4)

Gene expression profiling of gliomas has been studied to predict patient survival [12]. We analyzed the relationship between PLCβ1 gene expression using probe 213222_at and patient survival information in the REMBRANDT cohort. Glioma are stratified into groups based on PLCβ1 expression: 103 cases in the intermediate group, 226 cases in the downregulated (≥2X) group, and 0 cases in the PLCβ1 upregulation (≥2X) group. When analyzing PLCβ1 expression level in glioma subclass, astrocytoma cases are stratified into intermediate (*n* = 48) and downregulated (≥2X) groups (*n* = 57); there is no upregulated group (Fig. 5a, b). Log-rank test results show significant differences between the intermediate PLCβ1 group and the PLCβ1 downregulated groups for glioma (*p* = 3.0E-09) and its subclass astrocytoma (*p* = 2.0E-04) in a Kaplan-Meier survival plot. When PLCβ1 expression is further stratified as 3X, 4X, 5X, 6X, and 7X downregulation in glioma cases, log-rank tests still show significant differences between intermediate and downregulated groups (Table 4). When PLCβ1 expression is stratified by 3X downregulation in astrocytoma cases, the survival curve is still significantly different in the intermediate group compared to the downregulated PLCβ1 groups (*p* = 0.018) (Table 4). GAPDH expression is normalized internally among all cases (Fig. 5c, d).

Similar to a Kaplan-Meier survival curve for samples of differential PLCβ1 gene expression, subgroups can also be stratified by different ERBB4 expression. However, different levels of ERBB4 expression are only associated with survival in all glioma cases, but fail to show statistical significance among glioma subclass astrocytoma cases (Supplement Figs. S4A and S4B). We also conducted an analysis of a Kaplan-Meier survival plot for samples with differential Olig2 gene expression. Olig2 gene expression presents as bidirectional (up- and downregulation) changes in astrocytoma cases (*n* = 105), but there is no statistical significance in survival outcome among Olig2 gene upregulated (*n* = 62), intermediate level (*n* = 33), and downregulated (*n* = 10) subgroups (*p* > 0.05) (data not shown).

### PLCβ1 Expression Is Closely Associated with Patient Survival in the TCGA Cohort and GDS1815

**Fig. 6:**
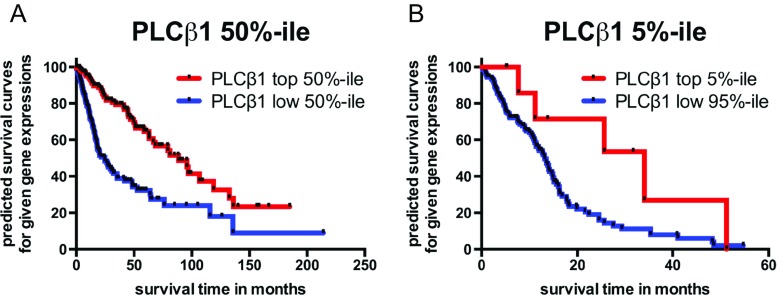
Kaplan-Meier survival curve for samples with differential PLCβ1 gene expression from the TCGA cohort. **a** Merged grade II–IV gliomas are stratified as high (*n* = 294) and low 50 % PLCβ1 expression (*n* = 293). There is statistical difference in survival between the two groups as indicated by the log-rank test (*p* = 4.8E-12). **b** GBM cases are stratified into the highest 5 % expression (*n* = 9) and the rest of 95 % (*n* = 142); there is statistical difference in survival between the two groups as indicated by the log-rank test (*p* = 0.039)

The TCGA cohort was also analyzed via a Kaplan-Meier survival curve based on PLCβ1 expression levels (RNA-Seq data). In a combination of all cases available from grades II to IV (*n* = 587), patients in the top 50 % of PLCβ1 expression group (*n* = 294) survived on average 88.7 months, significantly longer than those of the bottom 50 % percentile of PLCβ1 expression (*n* = 293) who survived on average 21.6 months (*p* = 4.8E-12; Fig. 6a). In analyzing data solely from GBM cases, which as a group contains the lowest PLCβ1 expression level, only subjects with top 5 % PLCβ1 expression (*n* = 9, mean = 25.7 months) survive significantly longer than the lower 95 % subjects (*n* = 142, mean = 13.2 months, *p* = 0.039; Fig. 6b). Results from analysis of the top 10 % versus the rest of the 90 %, and top versus bottom 50 % of PLCβ1 expressions, show no significant survival differences among GBMs (data not shown).

GDS1815 data can be used to evaluate the prognostic value of candidate genes because it contains information about patient age, survival, and tumor grades (Supplement Excel spread sheet). We found that the patients who died in fewer than 104 weeks (median duration = 55.4 weeks, range 3–102 weeks, *n* = 42) had a significantly lower level of PLCβ1 than those who survived 104 weeks and longer (median duration = 240.3 weeks, range 106–477 weeks, *n* = 35; Fig. 1d, *p* = 0.0035). Here we combined grade III and grade IV glioma for data analysis since each subgroup contains only a small number of cases.

### PLCβ1 Expression in Human Glioma Samples

**Fig. 7:**
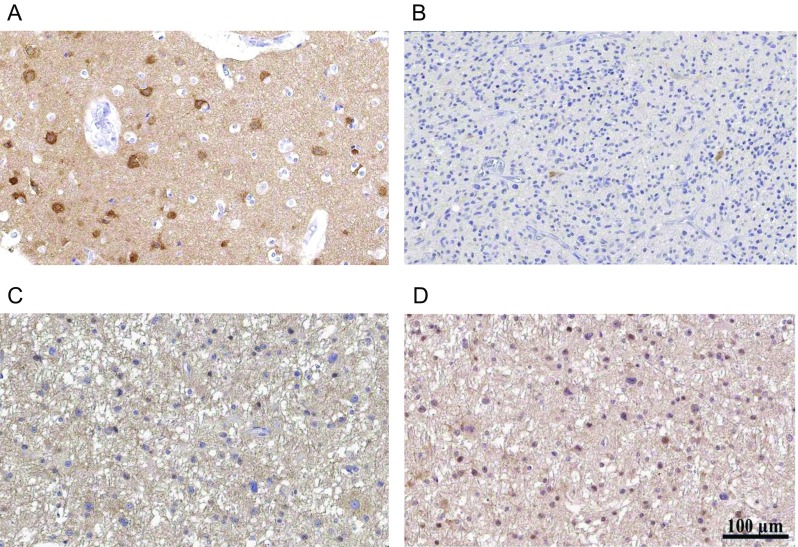
IHC images obtained from the HPA website. Antibody CAB005334 is used for images **a**–**c**, HPA034743 antibody is used for **d**. **a** PLCβ1 staining from normal cerebral cortex tissue of a 52-year-old female subject (patient ID—3740). **b** Negative PLCβ1 staining of glioma from a 75-year-old male subject (patient ID—2851). Both **c** and **d** are from one 32-year-old female subject (patient ID—122). **c** Cytoplasmic/membranous stain of PLCβ1 in glioma cells. **d** Nuclear stain of PLCβ1 in glioma cells. *Bar scale* equals to 100 μm

Images from the HPA website show that all four PLCβ1 antibodies stain cortical neurons of their cell bodies and neuropils, without staining glial cells in normal cerebral cortex (Fig. 7a). The majority of reports show negative PLCβ1 staining in glioma cells (Fig. 7b); both HPA057910 and CAB004275 antibodies yield negative staining in glioma cells in 11 glioma samples (data not shown). Only HPA034743 and CAB005334 show different intensities of positive staining in glioma tumor cells. Interestingly, from the same group, all HPA034743 staining is restricted to nuclei (Fig. 7c), while all CAB005334 staining is located at cytoplasm and cell membrane (Fig. 7d). We summarize the pathological report from these 11 patients and PLCβ1 staining in Table 3.

**Table 3:** Summary of pathology report about PLCβ1 antibodies staining in tumor cells from patients’ glioma IHC (source: Human Protein Atlas http://www.proteinatlas.org/)

Patient ID	Tumor Grade	Sex	Age	CAB005334, Santa Cruz Biotechnology			HPA034743, Sigma-Aldrich		
				Intensity	Quantity	Location	Intensity	Quantity	Location
122	Low	F	32	Weak	<25%	Cytoplasmic/membranous	Moderate	75%-25%	Nuclear
2892	Low	F	55	Moderate	<25%	Cytoplasmic/membranous	Weak	<25%	Nuclear
2895	Low	M	66	Negative	Negative	None	Weak	<25%	Nuclear
2909	Low	M	43	Weak	<25%	Cytoplasmic/membranous	Weak	>75%	Nuclear
45	High	M	72	Negative	Negative	None	Weak	<25%	Nuclear
2849	High	M	54	Weak	<25%	Cytoplasmic/membranous	Weak	<25%	Nuclear
2850	High	M	54	Negative	Negative	None	Weak	75%-25%	Nuclear
2851	High	M	75	Negative	Negative	None	Weak	75%-25%	Nuclear
2862	High	F	40	Negative	Negative	None	Weak	75%-25%	Nuclear
2891	High	M	49	Negative	Negative	None	Negative	Negative	None
2907	High	M	76	Negative	Negative	None	Weak	<25%	Nuclear

Note: data is ranked by tumor grade and then patient ID. IHC reports using the other two antibodies (HPA057910 and CAB004275) are not listed because all results are negative. *M* male, *F* female.

## Discussion

*Glioma*, the collective name for astrocytomas (grades I–IV), oligodendrogliomas (grades II–III), oligoastrocytomas (grades II–III), and ependymomas, is the most common primary brain tumor in adults [30]. Managing of high grade gliomas, especially GBM, remains a challenge in clinical practice due to the complexity of the multicellular background and genetic heterogeneity. We analyzed four independent microarray datasets from the NIH-maintained GEO databases, REMBRANDT, and the TCGA database and found that PLCβ1 gene expression (microarray and RNA-Seq) level is inversely correlated with gliomas’ pathological grades; it is a potential novel signature gene in subclassifying HGG into PN versus other subtypes, and its expression level also correlates with patients’ survival. Our study provides evidence for the first time that PLCβ1 is a candidate signature gene for PN subtype of HGG.

Previous research has highly recommended integrating molecular findings in current WHO classifications in order to generate a new histomolecular classification guideline [3, 8]. For instance, promoter methylation status of DNA repair enzyme *O* ^6^-methylguanine-DNA methyltransferase (MGMT), which affects patient chemosensitivity to temozolomide, has become a frequently requested laboratory test in neuro-oncology [4]. Patients with CpG island hypermethylation (G-CIMP) and isocitrate dehydrogenase 1 (IDH1) mutation have better prognoses [31, 32]. However, IDH1 mutation (∼10 %) and G-CIMP methylation positive (∼9 %) only account for a small percentage of primary GBM [33, 34]. IDH mutated glioma patients are significantly younger than those with IDH wild type, and IDH1/2 mutation is strongly associated with low grade astrocytomas. Paul Mazaris and his colleagues demonstrated that none of 31 GBM samples being tested harbored either IDH1 or IDH2 mutation [35]. Furthermore, studies demonstrated that prognostic signature genes that work well in long-term GBM survivors who have IDH mutations have no predictive value in IDH wild-type cases [36]. Thus, analysis of single gene mutations and/or epigenetic modifications are useful but have limitations in clinical practice. Novel classification of gliomas based on gene expression has shed light to better understanding of glioma pathogenesis in recent years. For example, Phillips et al. used 35 signature genes to classify 183 GBM samples into three subtypes: 31 % were PN, 20 % were proliferative, and 49 % were Mes subtypes. Clinical studies had demonstrated that patients with tumors displaying PN signature gene markers live longer, while those tumors have Mes signature genes had much shorter survival times (174.5 weeks for PN subtypes vs. 65 weeks for Mes subtypes) [11]. Per report by Verhaak and colleagues, patients with Mes subtypes respond favorably to standard treatment (temozolomide and radiation) and treatment significantly prolonged their survival, but PN subtypes do not benefit as much from these same treatments [10].

PLCβ has four isoforms, all of which can be detected in the brain. Only one or two isoforms, however, are predominantly expressed in neurons [16, 37]. PLCβ1, the predominant isoform in neurons, is involved in signal transduction in the cerebral cortex and hippocampus via its coupling to the muscarinic acetylcholine receptor [38]. It regulates neurogenesis since the knockout PLCβ1 gene affects cortical barrel formation in mouse model [18]. The PLCβ1 expression from the prefrontal cortex of developing human brain displays in an age-specific manner, suggesting that PLCβ1 is playing an important role in the differentiation and maturation of neurons in the developing brain [39]. Oligodendrocytes are also known to express intermediate levels of PLCβ1; astrocytes express the lowest level under culture conditions [20]. We theorize that this outcome is just inducible under culture conditions in vitro because freshly isolated glial cells are negative of PLCβ1 expression by PCR amplification [21, 22]. Thus, the finding that PLCβ1 expression was detected in glioma tissues by different methods such as microarray, RNA-Seq, and IHC indicates a very meaningful and possibly a pathological change. Furthermore, PLCβ1 microarray signal strength correlates well with other PN signature genes including DLL3, HEY2, Olig2, BCAN, and ERBB4 and negatively correlates with YKL-40 (Table 2 and Supplement Excel file), one commonly used mesenchymal cellular marker in dataset GDS1815. Grouping with the listed PN signature genes makes PLCβ1 another candidate signature gene for PN subtype glioma. Most of the microarray studies used three PLCβ1 probes; the original data generated by three probes in GDS1815 are highly correlated with one another (Supplement Excel spread sheet), which confirmed that these probes are of high specificity and binding efficacy; thus, users can be assured the data is of high reliability.

PLCβ1 gene expression inversely correlate with tumor grades (III and IV) and survival among glioma patients in GDS1815 dataset (Fig. 1), the presence of PLCβ1 transcripts are associated with PN subtype GBM from Mes (Figs. 1b and 2) and proliferative subtypes (Fig. 1c). Average PLCβ1 signal strength is also useful in separating grade III from grade IV HGG in GDS1962 (Fig. 4). Average PLCβ1 levels from normal tissue controls are significantly higher than those from low-grade tumors and its level is further downregulated in higher grade tumors, including astrocytomas and oligodendrogliomas in GDS1962 (Figs. 4 and S2). Because the TCGA database contains data for GBM as well as grade II/III gliomas, we merged these data and performed an analysis after removing batch effects. Normalized PLCβ1 expression inversely correlates with pathological grades of glioma: the higher the pathological grade, the lower the PLCβ1 expression (*p* < 0.0001, Fig. 2c). In contrast, GAPDH data showed no significant difference between grades III and IV samples, though grade II samples contain significantly lower amount of GAPDH than in HGG samples (*p* < 0.05; Fig. 2d).

A Kaplan-Meier survival curve of both REMBRANDT [glioma (Fig. 5a) and astrocytoma (Fig. 5b)] and TCGA data [grade II to IV merged (Fig. 6a) and GBM (Fig. 6b)] showed that different subgroups of PLCβ1 microarray/RNA-Seq expression levels correlate well with patient survival (*p* < 0.05). It is worth mentioning that only the top 5 % of PLCβ1 expression (*n* = 9) of GBM cases survived significantly longer than the rest of 95 % of PLCβ1 expression (*n* = 142) (Fig. 6b). However, the top 10 % and top 50 % PLCβ1 expression groups did not show survival benefits over the rest (data not shown). We believe that the GBM group contains the lowest level of PLCβ1 transcripts based on our analysis, but because there are many other factors affecting PLCβ1 signals (see discussion below), it is difficult to isolate a subgroup with statistically significantly “high” PLCβ1 expression than others. This reason may also explain why the REMBRANDT cohort analysis shows no statistical significant difference in survival among GBM cases (data not shown). ERBB4 expression level also shows unidirectional reduction as PLCβ1 does in the current analysis of the REMBRANDT cohort. Interestingly, a Kaplan-Meier survival curve demonstrates statistical significance among the stratified groups based on PLCβ1 expression in glioma and its subclass astrocytoma, while a Kaplan-Meier survival curve based on different ERBB4 expression levels only shows statistical significance among stratified glioma cases, but fails to show statistical significance among astrocytoma cases (Supplement S4). In GDS1815, when combining data from grade III and IV glioma, PLCβ1 level is significantly different between two groups of patients: <2 years (*n* = 48) vs. over 2 years survival (*n* = 35) (Fig. 1d). Thus, this unique application of PLCβ1 gene expression in predicting patient survival deserves further study in a large cohort from patients perspectively (Table 4).

**Table 4:** Kaplan-Meier survival analysis based on PLCβ1 gene expression level changes

	Stratification (by PLCβ1 downregulation)	Upregulated	Downregulated	Intermediate level	*p* value (downregulated vs. intermediate)
Glioma (*n* = 343)	2X	0	235	108	3.0E-09
	3X	0	163	180	2.5E-08
	4X	0	122	221	4.0E-09
	5X	0	86	257	3.9E-06
	6X	0	69	274	1.6E-05
	7X	0	54	289	7.9E-05
Astrocytoma (*n* = 105)	2X	0	57	48	2.0E-04
	3X	0	33	72	0.018

PLCβ1 level in gliomas was not only useful in the separation of low- and high-grade astrocytomas, but also has significant differences between grades II (low-grade) and III (high-grade) oligodendrogliomas (Fig. 4b and S4). Okamoto et al. [1] demonstrated that patients with oligodendrogliomas have the highest survival rate (78 % at 5 years, 51 % at 10 years), followed by those with oligoastrocytomas (70 % at 5 years, 49 % at 10 years), and fibrillary astrocytomas (65 % at 5 years, 31 % at 10 years). Survival of patients with gemistocytic astrocytomas was the poorest (16 % at 5 years and 0 % at 10 years). Confirming this observation, our data analysis showed that PLCβ1 signal in oligodendroglioma is significantly higher than that in pooled data of astrocytomas (Fig. S2B). A cellular study also demonstrated that PLCβ1 expression levels in oligodendrocytes were higher than that in astrocytes [20], a finding that may be useful in interpreting the clinical outcome whereby oligodendroglioma patients usually have a better prognosis than astrocytoma patients. Interestingly, GBM patients with an oligodendroglial component (*n* = 57) who survived longer (12 vs. 5.8 months; *p* = 0.006), comparing to 50 cases of other primary GBM, showed no difference in the frequency of common genetic defects, such as loss of heterozygosity of chromosome 1p/19q, MGMT promoter methylation, or IDH1 mutation [40]. We believe that PLCβ1 expression status may provide valuable information in the scenario when all known biomarkers are unrevealing. Separate from other known cancer biomarkers, which are usually upregulated at different stages of a disease, PLCβ1 expression level is the highest in normal tissues. Glioma samples show unidirectional reduction regarding PLCβ1 expression—the higher the pathological grade, the lower the level of PLCβ1 expression (microarray and RNA-Seq data). In addition, the REMBRANDT database only stratifies brain tumors as PLCβ1 “intermediate” and “downregulated” groups, in contrast to most of the other genes, which usually present as bidirectional changes. There is no subject in the “upregulated” group. This raises the possibility that overall PLCβ1 signal from glioma tumors is mainly determined by residual neurons, which harbor much higher levels of PLCβ1 than other cell types. The detected PLCβ1 level from gliomas is possibly a logical reflection of residual neurons’ PLCβ1 content, which will diminish as the tumor cells expand. PLCβ1 originating from glioma cells, if there is any, only contributes partially to measurable PLCβ1 gene expression.

Gliomas may originate from mixed cell types including oligodendrocytes and astrocytes as well as mesenchymal, neuroepithelial cells and cancer stem cells. However, the extent to which each cell type contributes to the overall level of PLCβ1 detected in patient tumor tissue has not been well studied. There are reports that glioma cell lines contain detectable PLCβ1; however, no information is available as to whether mesenchymal cells, despite being one of the common types of cells identified in glioma, express PLCβ1. Thus, it will be necessary to study cellular origins of PLCβ1 expression in gliomas to determine if different levels of PLCβ1 expression actually originate from oligodendrocytes, astrocytes, or even possibly mesenchymal cells versus normal and abnormal neuroprogenitor cells, or neurons.

Predominantly, cytoplasmic PLCβ1 expression occurs in neurons and their synapses and neuropils. As a result, abnormal PLCβ1 expression has been studied for its role in neurological diseases extensively such as seizure and epilepsy [41, 42]. In the analysis of The Catalogue of Somatic Mutations in Cancer, Mark G. Waugh demonstrated that the change in the number of gene copies of PLCβ1, as a component of phosphoinositide pathway, is involved in GBM; however, their conclusion that GBM cells *gain* PLCβ1 gene copies is different from our findings [43].

Accumulating evidence suggests a pathological role of PLCβ1 in glioma abnormality. Rodent studies showed that PLCβ1 expression is undetectable by PCR from freshly isolated astrocytes, but it can be detected in established astrocytoma cell lines and C6 rat glioma cell lines [15, 20–23]. Interestingly, PLCβ1 expression is inducible from primary cultured astrocytes when stimulated with lipopolysaccharide [22]. Pathological reports from the Human Protein Atlas show that PLCβ1 antibodies mainly stain neurons and neuropils in a normal brain without staining glial cells (Fig. 7a) [44]. However, some glioma samples are stained with PLCβ1 antibodies, both cytoplasmic and inside nucleus (Fig. 7c, d). These stains from human gliomas demonstrate that astrocytes can be induced to express PLCβ1 under pathological conditions.

PLCβ1 has two (a and b) isoforms which are different in their C terminals, and both isoforms contain a nuclear localization signal domain [45, 46]. Nucleus presence of PLCβ1 was demonstrated in cortical neurons of rabbit brain [15, 47], and evidence demonstrated that two isoforms have their preference in the cytosol and nuclear of C6 glioma cells, respectively [23]; PLCβ1 was also shown to be transited into the nucleus among C6 glial cell and Neuro2A cell (mouse neuroblastoma cell line) under stimuli [48]. Inside the nuclear, PLCβ1 is one of the key molecules that regulate nuclear inositides, and latest research concludes that nuclear inositides are independently regulated and nuclear inositol lipids themselves can modulate nuclear processes, such as transcription and pre-mRNA splicing, growth, proliferation, cell cycle regulation, and differentiation [49].

There have been no study found PLCβ1-mediated signaling in gliomas, and we speculate that PLCβ1 may play a role in glioma tumorigenesis. It has been demonstrated in hematological diseases including myelodysplastic syndromes and leukemia [50, 51] and other neurological diseases [52]. PLCβ1 and phosphoinositide 3-kinase (PI3K) share the common substrate PIP2. Thus PLCβ1’s activity regulates the availability of cellular PIP2 content in theory. Upon stimuli, PI3K converts PIP2 to phosphatidylinositol 3,4,5-trisphosphate (PIP3), which leads to cascade downstream reaction involving AKT and mTOR. Evidence exists that PLCβ1 interconnected with PI3K/AKT/mTOR signaling in solid tumors and hematological malignancies [49, 53]. In a study of pro-B-lymphoblastic cell, b isoform PLCβ1 promotes cell survival by affecting AKT activation, cyclin E expression, and caspase cleavage [54]. In addition, our analysis demonstrates that PLCβ1 gene expression level correlates the best with glioma PN signature gene ERBB4. ERBB4 protein is a tyrosine-protein kinase and a member of the epidermal growth factor receptor subfamily, which contributes to glioma pathogenesis. At the protein level, typical ERBB4 signaling in the central nervous system involves downstream PLC and PI3K-AKT activation; cleaved intracellular fragment translocates into nucleus and regulates gene transcription [52]; cleaved ERBB4 protein also plays an important role in regulating the timing of astrogenesis in the developing brain [55]. It is worth to mention that ERBB4 expression is mainly restricted within neurons [56, 57] like PLCβ1 does among mature rodent brains, not commonly expressing a high level among astrocytes. It is possible that ERBB4 and PLCβ1, as neuron predominant proteins, contribute to glioma tumorigenesis or progression.

As shown in this study, the PLCβ1 signal strength varies among samples and in the same subgroups. Since microarray signal strength and RNA sequencing reflects mRNA content in transcriptome, mRNA stability or instability, different amount of normal tissue mixed into the tumor samples can contribute to this. Factors such as tissue sampling and storage condition, sample processing time lag from initial tissue harvesting, treatment regimen (chemo- and radiotherapy) and time length, primary versus recurrent tumor, tumor pathological grades, and classification could all contribute to a wide variation of PLCβ1 expression detected. In addition, PLCβ1 microarray probes only target and bind to common cDNA region of both a and b isoforms; however, two isoforms are different in their C terminals; thus, microarray data based on current PLCβ1 probes could not differentiate the a or b isoform. If the transcription of one PLCβ1 isoform is predominant isoform, the minor component of overall PLCβ1 signal may not contribute to our conclusion reached in this study: the higher the glioma grade, the lower the PLCβ1 expression. It will be possible for one isoform to gain its signal strength along with pathological grades. More research is certainly needed to substantiate this observation.

**Fig. 8:**
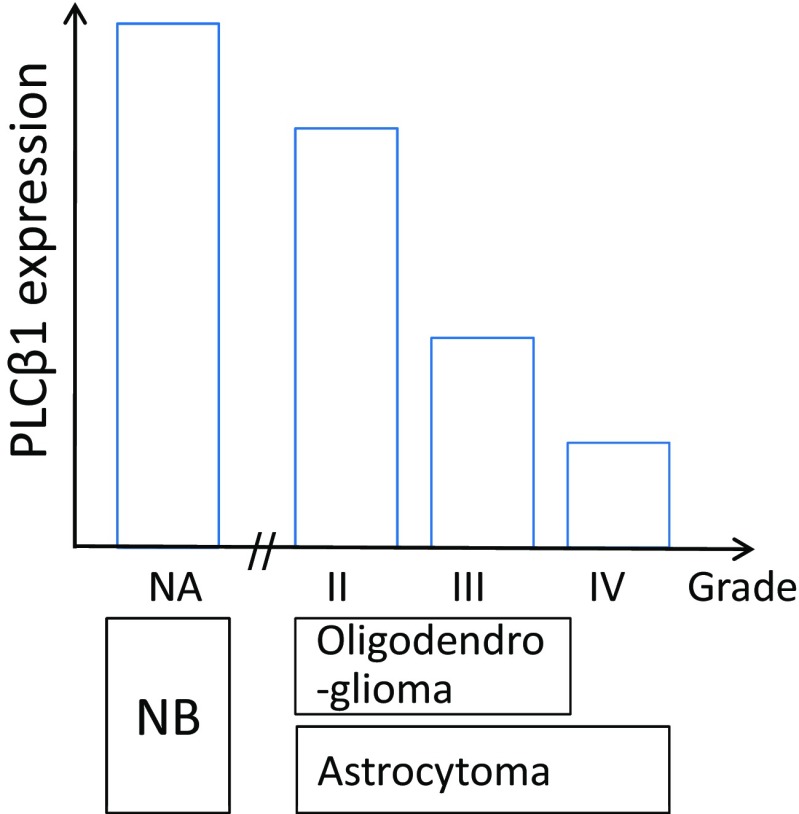
Graphic diagram displays a relationship between glioma PLCβ1 expression and pathological grades. Normal brain (*NB*) expresses the highest level of PLCβ1. Among the astrocytomas, the higher the pathological grade (II to IV), the lower is the PLCβ1 expression. This inverse relationship also applies to oligodendrogliomas (grades II and III)

In summary, our data analyses of TCGA and four independent GEO datasets revealed an association between differential expression of PLCβ1 and glioma pathological grades (Fig. 8). PLCβ1 is a potential novel signature gene for PN subtypes in molecular classification of HGG because its gene expression correlates with known PN subtype signature genes; its inducible property in glial cells further supports its role as a biomarker in glioma classification. Kaplan-Meier survival curves based on differential PLCβ1 gene expression from the REMBRANDT and TCGA cohorts also demonstrate that high level of PLCβ1 expression associates with patient’s long-term survival. Quantitative PLCβ1 microarray/RNA-Seq result(s) could be incorporated with current molecular assay tools to supplement tradiational neuropathology in classifying HGG subtype(s), potentially contributing to glioma patient prognosis and measuring therapy effectiveness.

## Electronic Supplementary Material

Below is the link to the electronic supplementary material.

ESM 1 (XLS 103 kb)

ESM 2 (XLS 139 kb)

ESM 3 (DOC 1459 kb)
