# Supplementary material for: Phospholipase C Beta 1: a Candidate Signature Gene for Proneural Subtype High-Grade Glioma
Source: Mol Neurobiol. 2015 Nov 28;53(9):6511–25. doi: 10.1007/s12035-015-9518-2 (PMC5085994; doi:10.1007/s12035-015-9518-2)
Supplement: Supplementary file 3 — (DOC 1459 kb) [file 12035_2015_9518_MOESM3_ESM.doc]

**Supplement data**

A B


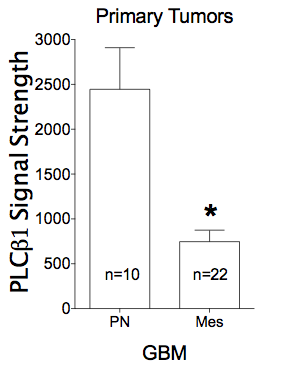

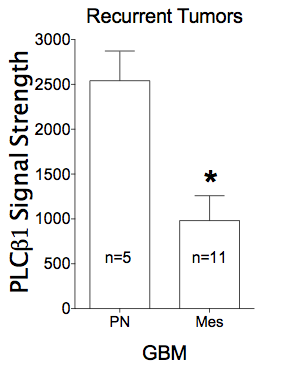


Figure S1. GBM are further stratified into primary *(A)* and recurrent status *(B)* in GDS1815 dataset, PLCβ1 microarray signal strengths were both significantly lower in Mes subtype than its level from PN subtype, p=0.0052 and 0.0051 respectively for analyzing primary and recurrent tumors, *p<0.05.

A B


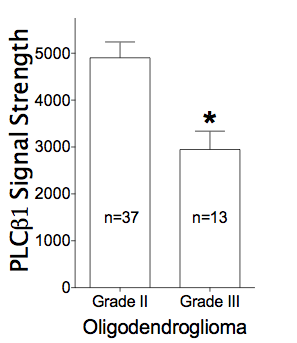

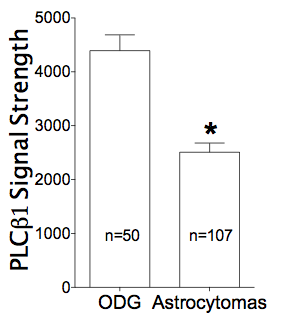


Figure S2. *A)* In GDS1962, PLCβ1 signal levels from grade II (n=37) oligodendroglioma are significantly higher than that from grade III oligodendroglioma (n=13), p=7E-5. *B)* Oligodendroglioma (ODG, n=50) average PLCβ1 signal was significantly higher than pooled data of astrocytomas (n=107), p=1.4E-6. * indicates p<0.05.

A


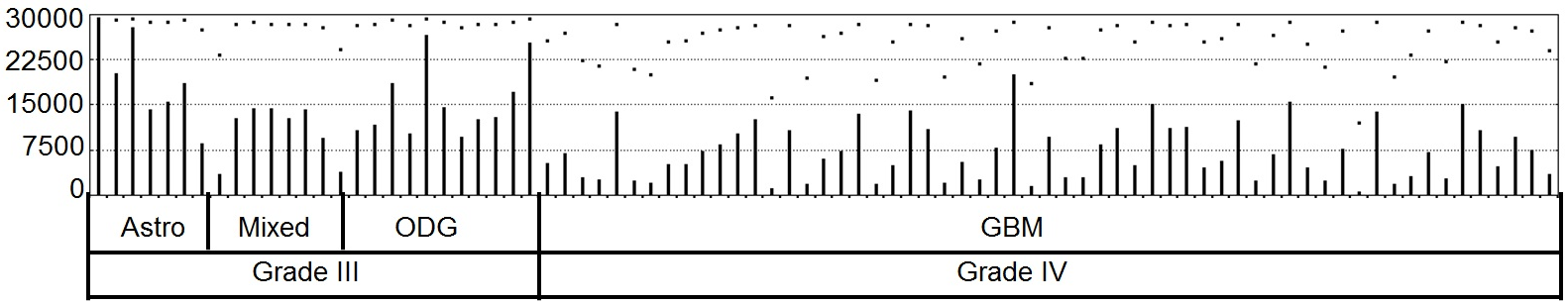


B


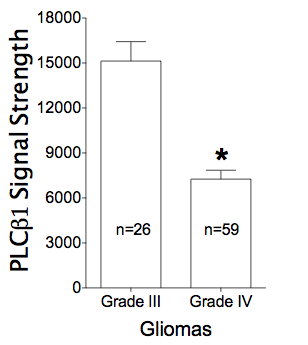


Figure S3. Analyzing PLCβ1 expression profile of GDS1975 dataset. *A)* Original microarray data, generated by probe 213222_at, copied from NIH website, with minor modification. Astro, Mixed and ODG stands for anaplastic astrocytoma, anaplastic mixed oligo-astrocytoma and oligodendroglioma respectively. *B)* PLCβ1 signal strength from grade III (n=26) are significantly higher than its level from grade IV gliomas (n=59), p=3E-6. * indicates p<0.05.

A B


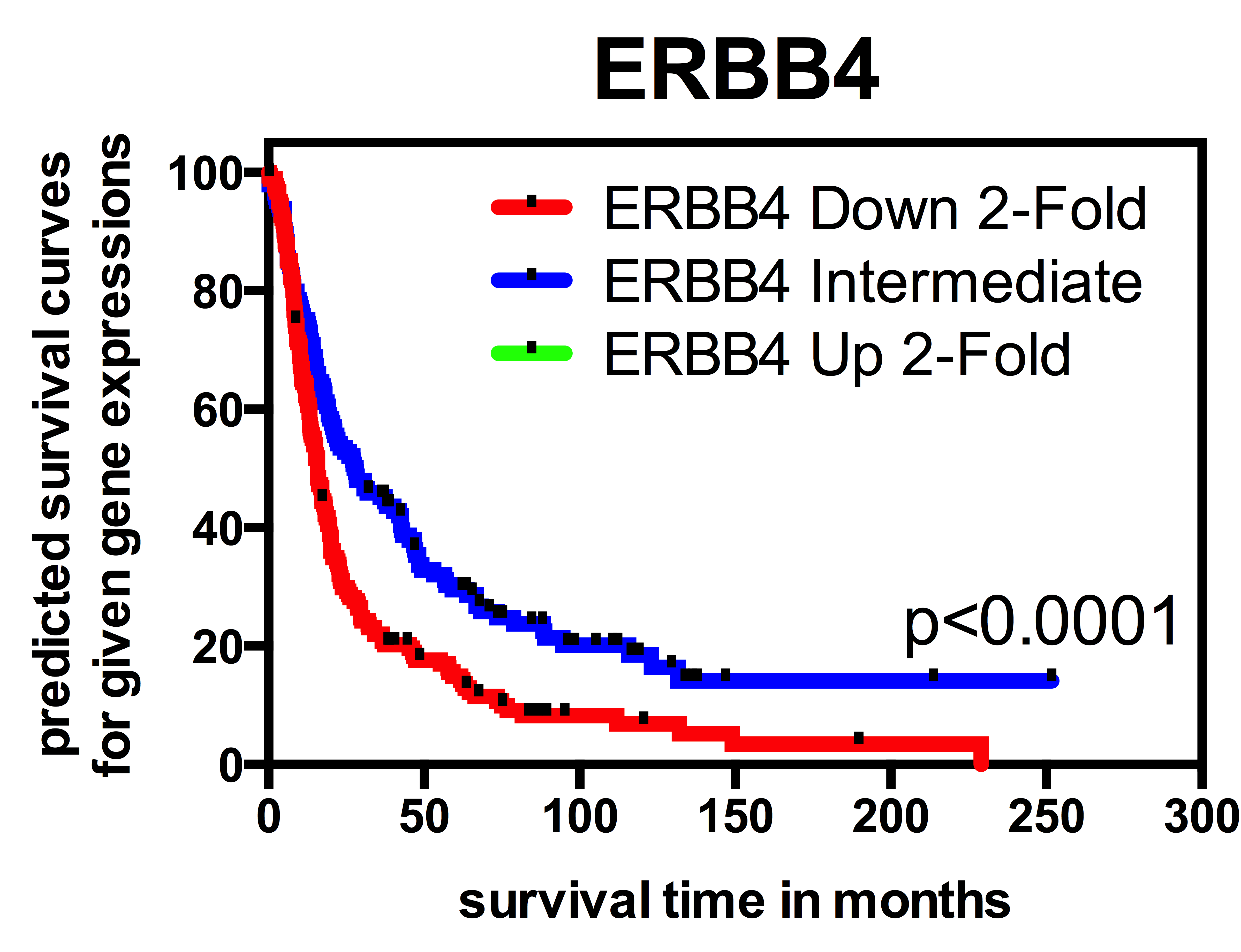

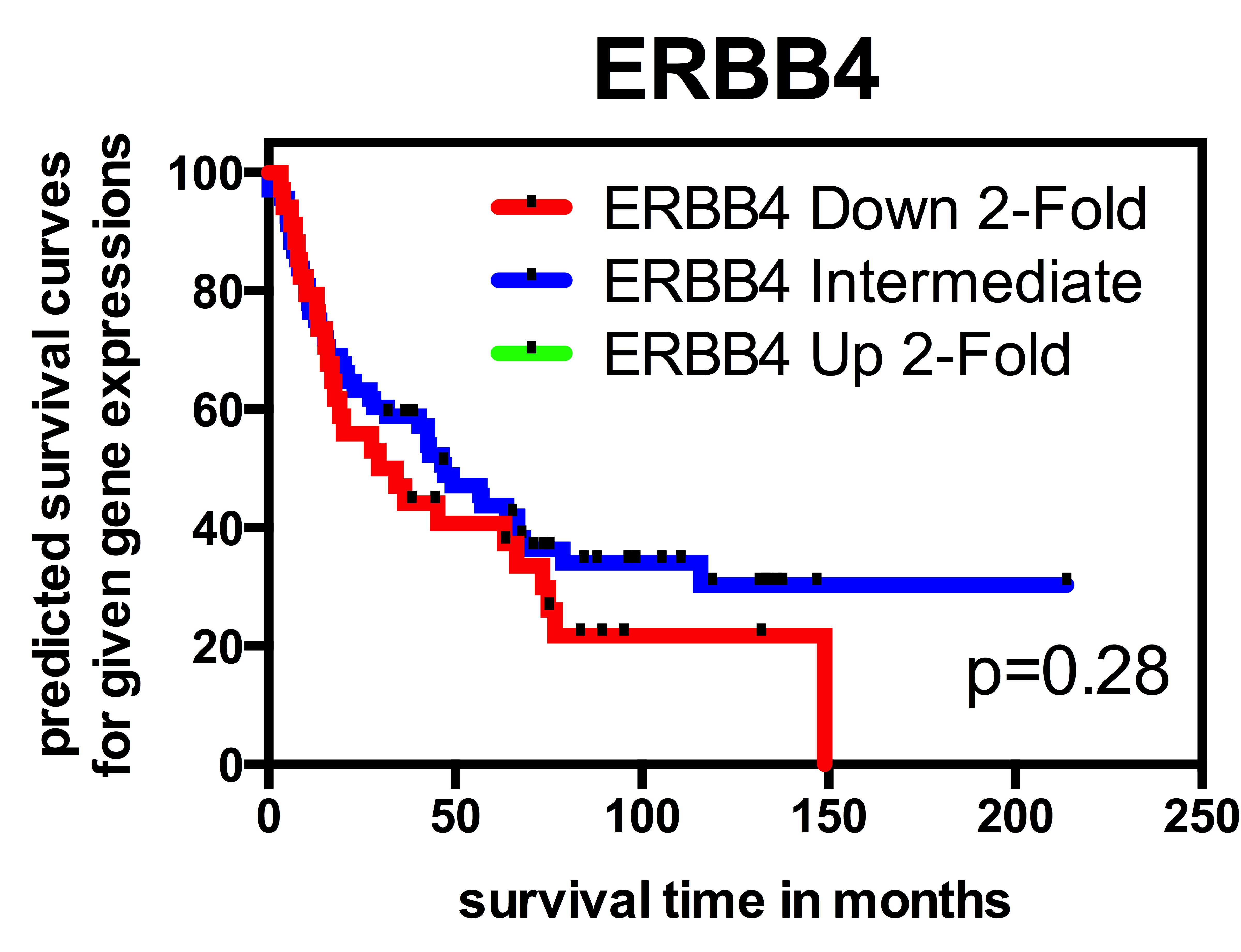


Figure S4. Kaplan-Meier survival plot for glioma and astrocytoma samples with differential ERBB4 gene expression, raw data are extracted from REMBRANDT cohort. *A)* ERBB4 down-regulated glioma patients (n=182) survived significantly shorter times than cases of intermediate level ERBB4 (n=147), per Logrank test, p<0.0001. *B)* ERBB4 down-regulated astrocytoma patients (n=34) show no statistically significant difference in survival time from cases of intermediate level ERBB4 (n=68), per Logrank test, p=0.28. Both databases contain no cases being stratified as up-regulated ERBB4 expression (≥2X).

GENE EXPRESSION CORRELATION and SURVIVAL DATA ANALYSIS for GDS1815 dataset are attached as supplement.

Data_1: title: GDS1815-Gliomas-Correlation-Analysis. Excel file contains data of gene expression levels is provided. Original data is directly copied from website under each probes, and aligned by patient ID. Genes listed include PLCβ1 (3 probes), PLCβ4, GFAP, AKT, DLL3, HEY2, ASCL1, Olig2 (2 probes), BCAN (3 probes), ERBB4 (2 probes) and YKL_40 (3 probes). Data generated from 3 PLCβ1 probes correlate each other very well, with a correlation coefficient scores over 0.70. DLL3, HEY2, Olig2, BCAN, ERBB4 and YKL_40 are known signature genes listed by other study.

Data_2: title: GDS1815-Gliomas-Survival-Analysis. Excel file contains survival data and PLCβ1 gene expression is provided. Original survival data is directly copied from each study subject’s profile, and aligned with other parameters by patient ID. Data is ranked by “Exact survival” time, analyses is based on two groups separated by 2 years (204 weeks) survival time: n=42 for patients survived less than 2 years, n=35 for patients survived over 2 years; there are 23 cases with no survival information provided in original dataset.
